# Results in Hip-joint Amputations

**Published:** 1868-08

**Authors:** 


### Results in Hip-joint Amputations.

The attention of our readers will be attracted to the correspondence from Dr. George A. Otis, Assistant Surgeon, and Brevet Lieut. Col. U. S. A., in reply to the communication we received and published in the February number of our Journal, from Dr. Paul F. Eve. The review of Dr. Eve's report in our January number contained some inaccuracies, but in the main appears perfectly fair and just. The idea of claiming for the Army Surgery of the South, any better results than for that of the North, is simply absurd. If Dr. Eve designed to give any such impression, or entertained any opinions of that sort, we hope he will be glad of the correction; but we presume he did not intend to give such an idea to his readers. This communication from Dr. Otis was designed for our last number, but was miscarried and reached us too late.

